# Respiratory syncytial virus prevention in infants and equity in Latin America and the Caribbean: from evidence to action

**DOI:** 10.1016/j.lana.2025.101316

**Published:** 2025-11-24

**Authors:** María L. Avila-Aguero, Luiza Helena Falleiros-Arlant, José Brea, Carlos Espinal, Flor Muñoz

**Affiliations:** a Hospital Nacional de Ninos, San José, Costa Rica; b Universidade Metropolitana de Santos, Sao Pablo, Brazil; c Facultad de Salud del Instituto Tecnológico de Santo Domingo, Santo Domingo, Dominican Republic; d Florida International University, Miami, USA; e Texas Children's Hospital, Houston, USA

Respiratory syncytial virus (RSV) is the leading cause of acute lower respiratory tract illness in infants worldwide, responsible for more than 100,000 deaths annually in children under five, most of them in low- and middle-income countries.^1^ In Latin America and the Caribbean (LAC), where the majority of disease likely occurs in the community, RSV-associated bronchiolitis and pneumonia are also important causes of pediatric hospitalization, indication for oxygen therapy, and admission to intensive care units.^2^ The burden of morbidity and mortality is highest among the most vulnerable: preterm infants, children with underlying chronic diseases or altered immunity, and those living in disadvantaged or remote areas.^1^, ^2^, ^3^

The recent availability of two highly effective prophylactic strategies, maternal vaccination during pregnancy (RSVpreF bivalent vaccine) and long-acting monoclonal antibodies (nirsevimab and clesrovimab) administered to infants from birth through the first months of life, represents a historic opportunity to prevent severe RSV disease in the region.^4^, ^5^, ^6^ However, their implementation must be framed within the principles of the children's right to health and guided by a strong commitment to equity and health justice.

LAC is becoming a reference region for RSV prevention, with countries adopting diverse strategies adapted to local contexts. Chile implemented a universal approach with monoclonal antibodies, protecting all newborns with a dose at birth, infants under six months of age as a catch-up dose, and high-risk children up to two years of age, with clear public health impact and broad community acceptance.^5^ Argentina prioritized maternal vaccination as its main strategy, showing effectiveness in preventing hospitalizations and severe disease consistent with the efficacy reported in pre-licensure clinical trials and a more modest effect on healthcare utilization.^4^ Paraguay introduced monoclonal antibodies at birth and in the first months of life and then expanded coverage during its second season, while Colombia, Uruguay and Costa Rica are initiating maternal immunization programs. Collectively, these experiences reflect regional leadership in supporting the prompt implementation of RSV prevention strategies and the evaluation of their impact, as well as a commitment to evidence-based decision making to ensure health equity by addressing local needs.^7^^,^^8^

The challenges to the region are not scientific or technical, but rather those fundamentally associated with equity and justice. Who will have access to these new immunizations? When? Will the programs be cost-effective? Will new strategies reduce existing inequities or perpetuate them? Health equity is not merely the fair distribution of interventions; it is ensuring that child survival does not depend on birthplace or socioeconomic status. In a region with broad inequities like those present in LAC, the risk is clear: unequal access will widen the gap between rich and poor, which must be avoided.

Disadvantaged children are already at greater risk of hospitalization and death from RSV due to underlying health conditions such as malnutrition, and barriers in accessing healthcare, in addition to existing weak healthcare systems. A fragmented rollout where some receive maternal vaccine or infant antibodies while others do not would deepen these injustices. The introduction of RSV immunizations must be equity-driven from the outset.

The region has demonstrated that solidarity is possible. For decades, the Pan American Health Organization's Revolving Fund has ensured that all countries, large or small, gain access to vaccines at affordable prices. The successful introduction of pneumococcal and rotavirus vaccines showed that regional consensus, supported by fair financing and strong disease surveillance, accelerates adoption.^9^^,^^10^ Conversely, when financing was uncertain or evidence was incomplete, delays led to preventable suffering. Support from manufacturers to ensure that the cost of immunizations is affordable to the region is critical. These lessons apply directly to RSV prevention.

Supporting evidence must be inclusive to guide decisions. Without robust and stratified surveillance, the realities of vulnerable populations remain invisible. RSV surveillance systems must be supported to capture disease impact and safety data in all groups at risk, as well as geographic and socioeconomical issues particular to each country. Cost-effectiveness analyses must integrate equity as a central criterion, not just efficiency. Pediatric societies such as the Latin American Society for Pediatric Infectious Diseases (SLIPE) and the Latin American Society of Vaccinology (SLV) have underscored the importance of participatory approaches, with evidence generated for and with communities, in addition to guidance from local experts and regulatory bodies.^7^^,^^8^

To ensure equity in RSV prevention, based on available resources, countries should adopt either universal administration of monoclonal antibodies offered to all newborns and infants entering their first RSV season (through 7 months of age), as well as to high-risk infants through their second RSV season; or routine maternal vaccination in every pregnancy complemented by targeted monoclonal antibody use. With maternal immunization as the primary strategy, monoclonal antibodies should be available for newborns and infants through 7 months of age whose mothers were not vaccinated, newborns and infants through 7 months of age born within 14 days of maternal immunization, preterm infants, and those with underlying conditions that increase the risk of complications from RSV. The timing of administration of maternal and infant immunizations should be tailored to the local epidemiology, varied climates and RSV seasonality. In regions with year-round circulation of RSV, year-round immunization rather than seasonal would be more appropriate. Year-round administration also facilitates access to immunizations, and could increase coverage. A comprehensive and inclusive approach is essential to achieving justice in access and outcomes. The expected decrease in disease burden and healthcare costs supports investment in such integrated strategies.^11^

In addition to financing, mechanisms of solidarity, including pooled procurement, tiered pricing, and technical cooperation, are essential to a successful implementation. Cultural relevance, clear communication, and patient trust are equally decisive for acceptance. Without these elements, even the best immunizations may fail to reach those who need them most.

The introduction of preventive strategies for RSV in early life in LAC can be transformative for children's health and survival in the region. The legacy will depend on today's decisions to support strengthening disease and safety surveillance, conducting economic analyses with an equity perspective, leveraging manufacturer contract agreements and the PAHO Revolving Fund to negotiate fair prices, ensuring complementary access to maternal vaccines and infant monoclonal antibodies, prioritizing vulnerable populations in early implementation, and involving civil society to sustain policies rooted in justice and solidarity.

The science is robust; the tools exist. What must not be lacking is the political and moral will to ensure that every child, regardless of birthplace or socioeconomic condition, receives protection. Prevention of RSV in infants presents a historic opportunity for justice and equity in child health in LAC, and the responsibility lies in our hands.

## Contributors

MLAA conceived the idea for this Comment and drafted the first version. All authors contributed to the design, critically revised the manuscript for important intellectual content, and approved the final version.

## Declaration of interests

FMM: member of DSMB for RSV and GBS vaccines, Pfizer; Consultant Sanofi, Astra Zeneca and Merck. MLA has received fees for vaccine lectures and for vaccine implementation strategies and vaccine hesitancy. All other authors declare no competing interests.
